# YAP1 regulates ABCG2 and cancer cell side population in human lung cancer cells

**DOI:** 10.18632/oncotarget.13686

**Published:** 2016-11-29

**Authors:** Yuyuan Dai, Shu Liu, Wen-Qian Zhang, Yi-Lin Yang, Phillip Hang, Hui Wang, Li Cheng, Ping-Chih Hsu, Yu-Chen Wang, Zhidong Xu, David M. Jablons, Liang You

**Affiliations:** ^1^ Thoracic Oncology Laboratory, Department of Surgery, Comprehensive Cancer Center, University of California, San Francisco, CA, USA; ^2^ Department of Thoracic Surgery, Beijing Chao-Yang Hospital, Affiliated with Capital University of Medical Science, Beijing, People's Republic of China

**Keywords:** ABCG2, YAP1, side population, cancer stem cells, lung cancer

## Abstract

A small population of cancer cells called cancer-initiating cells or cancer stem cells (CSCs) are involved in drug resistance, metastasis, and cancer relapse. Finding pathways that regulate CSC is very important for clinical therapy. ATP-binding cassette sub-family G member 2 (ABCG2) plays a role in side population (SP) cell formation and contributes to chemotherapy resistance in common forms of cancer. Yes-associated protein 1 (YAP1) is a major transcriptional effector of the Hippo pathway, which plays important roles in organ size control and tumorigenesis. In this study, we found ABCG2 and YAP1 were both overexpressed in lung cancer SP cells. Disruption of YAP1 expression by siRNA attenuated the expression of ABCG2 transcript and significantly reduced the percentage of SP cells and sphere formation in lung cancer cells. Overexpression of YAP1 in lung cancers led to an increase in ABCG2 expression and increased the percentage of SP cells. However, overexpression of YAP1 in purified non-SP cells did not increase ABCG2 expression and the percentage of SP cells, which may be due to the inhibition of YAP activity through phosphorylation. YAP1 directly transcriptionally regulated ABCG2 by binding to the promoter of ABCG2. Moreover, the YAP1 inhibitor verteporfin and YAP1 siRNA downregulated ABCG2 level through inhibition of YAP1 in lung cancer cells and sensitized them to the chemotherapy drug doxorubicin. Our study adds a new function for YAP1 that may be relevant to drug resistance and cancer therapy through regulation of ABCG2 and side population cell formation in lung cancer.

## INTRODUCTION

Accumulating evidence indicates that tumors are a heterogeneous mixture containing mostly non-stem cells and a small subpopulation of cancer stem cells (CSCs) [1]. Despite the self-renewal and tumor initiation ability of CSCs, they can also export certain toxic compounds resistant to many chemotherapeutic agents and cause tumor relapse [2]. The compound efflux ability of CSCs comes from the increased expression of ATP-binding cassette (ABC) transporters within the cell membrane and serves as the basis of an important flow-cytometry-based cell-sorting assay called the side population (SP) assay [3–6]. The SP assay is characterized by the differential potential of cells to efflux a DNA-binding dye, Hoechst 33342. The high efflux ability of CSCs leads to a low retention of Hoechst 33342 fluorescent signal in these cells, which reside at the low-left corner in flow cytometry analysis and thus are also known as side population (SP) cells or cancer stem-like cells [7]. The SP assay has emerged as a promising method to identify stem cells and determine drug efficacy in killing CSCs because it quantitatively analyzes the relative number (%) of stem-like cancer cells in the overall cancer cell population [8, 9].

In lung cancer cells, ATP-binding cassette sub-family G member 2 (ABCG2), an ABC transporter member, is responsible for SP formation [8, 10]. ABCG2 contributes to chemotherapeutic drug resistance in lung cancer treatment and appears to be a predictor of survival in patients with advanced non-small cell lung cancer [11]. SP cells sorted out from lung cancer cells also demonstrate a greater tumorigenic capacity than non-SP cells [5]. Consequently, determining the molecular mechanism that controls ABCG2 expression in lung cancer is very important for developing more effective therapy that can downregulate ABCG2 and lead to eradication of cancer stem cells.

The Hippo pathway was initially defined in *Drosophila*. Because mutations in components of this pathway lead to tissue and organ overgrowth, the Hippo pathway is considered as a tumor suppressor pathway. In mammals, the Hippo pathway consists of a conserved core kinase cascade that includes serine/threonine kinases MST1/2 (mammalian Ste2-like kinases 1/2) and LATS1/2 (large tumor suppressor kinase 1/2). In humans, when the Hippo pathway is activated MST1/2 phosphorylates and activates Lats1/2 kinase functions to inactivate Yes-associated protein 1 (YAP1) by directed phosphorylation on YAP1 Ser 127. Phosphorylated YAP1 is sequestered in the cytoplasm via binding to 14-3-3 and results in degradation. Conversely, dephosphorylated YAP1 localizes in the nucleus and acts mainly through TEAD family transcription factors to induce gene expressions that promote cell proliferation and organ growth. The other major effector of the Hippo pathway, called TAZ (transcriptional coactivator with PDZ binding motif), is regulated by LATS1/2 and acts with TEADs in a similar manner to that of YAP1 [12–14]. TEAD transcription factors mediate genome-wide YAP1 chromatin-binding [15]. The known consensus motif for TEAD is CATTCC [14]. YAP1 overexpression due to amplification of the YAP1 gene, loss of Hippo signaling by mutation, and/or down-regulation of core Hippo components have been found in many cancers [16]. YAP1 overexpression also contributes to self-renewal and tumor-initiation capacities in cancer stem cells [17, 18]. YAP1 also reportedly contributes to promoting resistance to anticancer drugs in different cancers like ovarian cancer and hepatocellular carcinoma cells [19–22]. Verteporfin (trade name Visudyne) has been identified as a YAP1 inhibitor and can be readily used to study the effects of YAP1 [23, 24]. However, the relationship between YAP1 and ABCG2 and YAP1 regulation of cancer cell side population in lung cancer have never been reported.

In this study, we asked whether YAP1 regulates ABCG2 in lung cancer cells. To answer this question, we investigated whether ABCG2 is a direct downstream target of YAP1 and the potential therapeutic advantage of this transcriptional regulation if it was confirmed.

## RESULTS

### YAP1 activity and ABCG2 mRNA and protein levels are higher in SP cells than in non-SP cells

**Figure 1:**
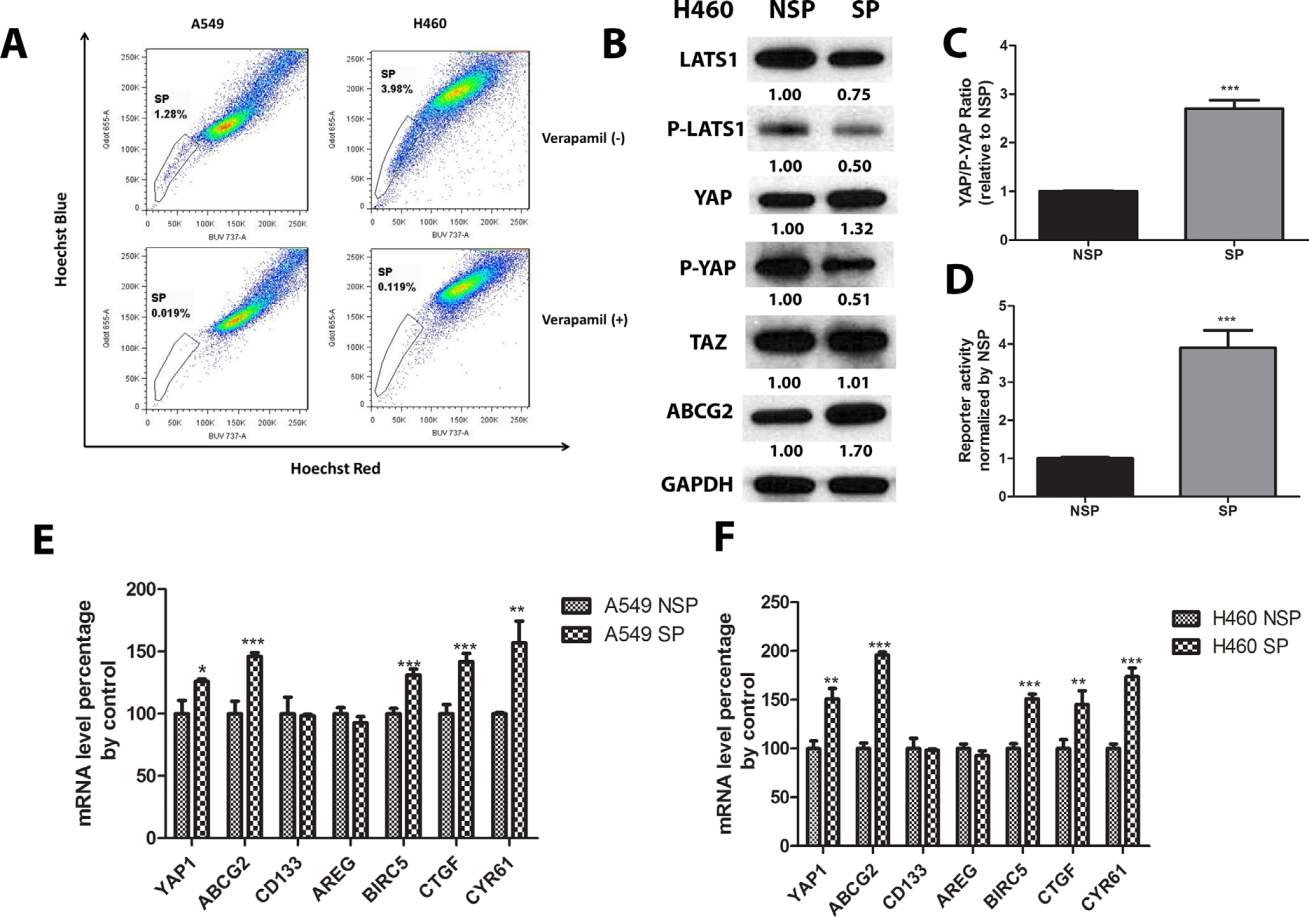
YAP1 activity and ABCG2 mRNA and protein levels are higher in SP cells than in non-SP cells (**A**) Flow cytometry analysis of SP cells in A549 and H460 shows portion of SP cells in A549 and H460; (**B**) Western blot analysis of LATS1, P-LATS1 (Thr1079), YAP1, P-YAP1 (Ser127), TAZ, and ABCG2 protein level in H460 non-SP cells and SP cells. GAPDH was detected as a loading control. Band intensity was analyzed with ImageJ software and normalized with the intensity of GAPDH band. (**C–D**) Bar graph showing YAP/P-YAP ratio in purified SP and non-SP (NSP) of A549 and H460. (**E–F**) qPCR analysis of mRNA level of YAP1, ABCG2, CD133, AREG, BRIC5, CTGF, and CRY61 in non-SP cells and SP cells of H460 and A549. Data are representative of at least three independent experiments. Error bars indicate the standard deviation of triplicate qPCR data. *P < 0.05, **P < 0.005, ***P < 0.001.

In order to analyze ABCG2 and YAP1 in SP cells, we first sought to determine whether SP cells were present in human NSCLC cell lines A549 and H460 and to sort out SP cells and non-side population (non-SP) cells for analysis. The SP cells were detected based on their ability to exclude Hoechst 33342 dye and appeared as a distinct tail at the bottom-left corner in the flow cytometry plots. When the cells were pretreated with the ABC transporter inhibitor verapamil, the tail disappeared or faded out. The position where the tail disappeared was used as a control to gate the area of SP cells. The A549 and H460 cell lines respectively contained 1.28% and 3.98% SP cells. After the cells were treated with verapamil, these percentages decreased to 0.019% in A549 cells and 0.119% in H460 cells (Figure 1A). Western blot analysis of protein in H460 SP and non-SP cells showed lower levels of LATS1, phosphate-LATS1 and phosphate-YAP1 (S127), but higher total YAP1 and ABCG2 levels in H460 SP cells than in non-SP cells (Figure 1B). Around 3-fold YAP/P-YAP ratio in SP cells of in non-SP cells indicated higher YAP1 activity present in SP cells (Figure 1C), as did the higher level of GTIIC reporter activity in H460 SP cells than in non-SP cells (Figure 1D). The SP and non-SP cells in both NSCLC cell lines were then analyzed using Q-RT-PCR. In both cell lines, mRNA levels of ABCG2, YAP1 and YAP1 downstream genes including *BIRC5*, *CTGF* and *CYR61* were higher in SP cells than in non-SP cells except *CD133* and *AREG* (Figure 1E and 1F).

### Knockdown of YAP1 decreases ABCG2 expression, the percentage of SP cells and the number of spheres formed in A549 and H460 cells

**Figure 2:**
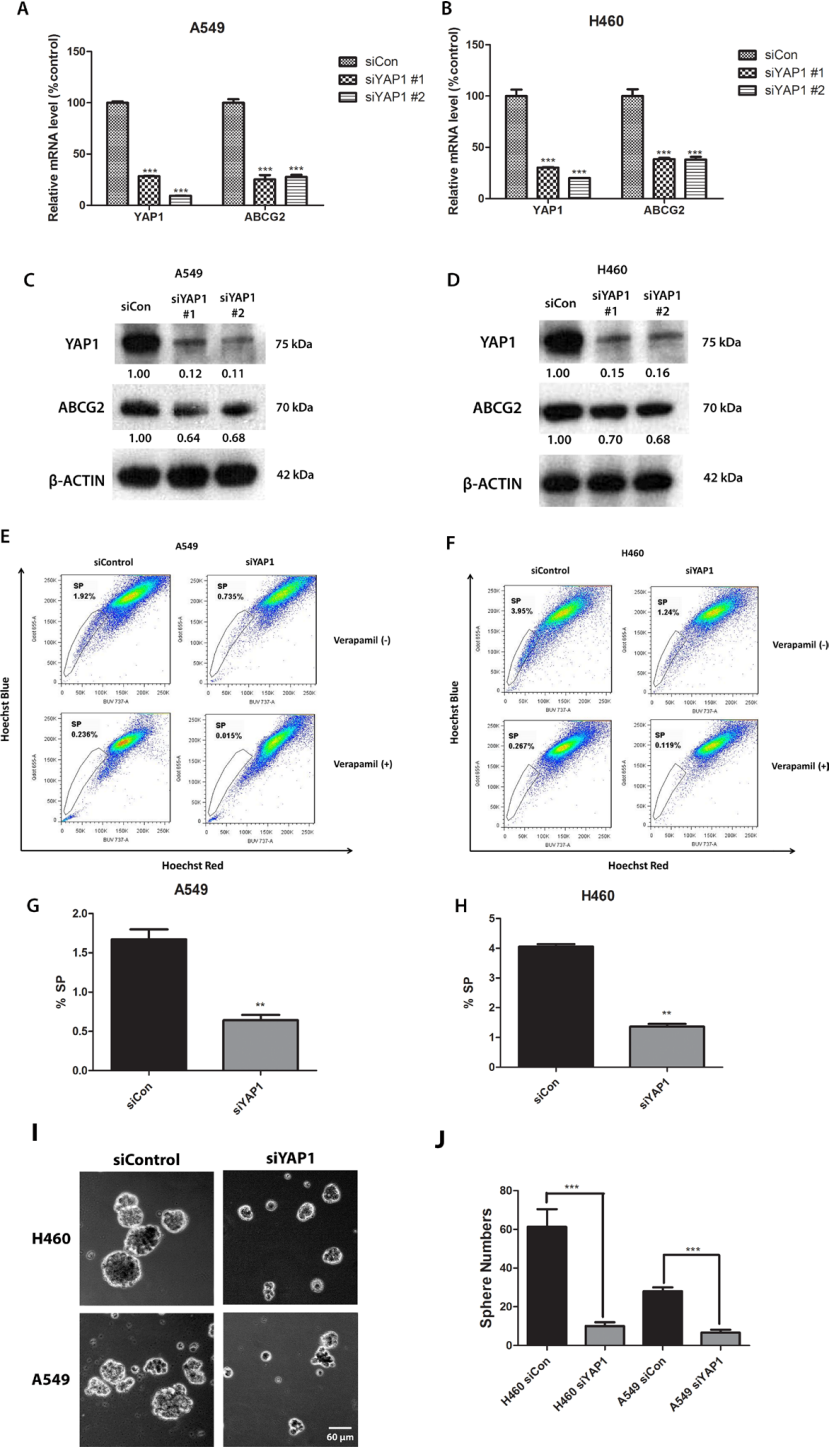
Knockdown of YAP1 decreases ABCG2 expression and the percentage of SP cells in NSCLC cell lines A549 and H460 (**A–B**) qPCR analysis of mRNA level of YAP1 and ABCG2 in H460 and A549 after YAP1 siRNA (siYAP1#1 and siYAP1 #2) treatment. (**C–D**) Western blot analysis of protein level of YAP1 and ABCG2 in H460 and A549 after YAP1 siRNA treatment. β -ACTIN was detected as a loading control. Band intensity was analyzed with ImageJ software and normalized with the intensity of β-ACTIN band. (**E–F**) Flow cytometry analysis of the SP cell portion in A549 and H460 after siYAP1 #2 treatment. (**G–H**) Bar graph showing the percentage of SP cells in A549 and H460 after siYAP1 #2 treatment. (**I**) Sphere formation analysis of H460 and A549 after control or YAP1 siRNA transfection. (**J**) Bar graph showing the number of spheres formed in H460 and A549 after control or YAP1 siRNA transfection. Data are representative of at least three independent experiments. Error bars indicate the standard deviation of triplicate qPCR data and SP assay data. **P < 0.005.

To investigate whether depletion of YAP1 influences ABCG2, we treated A549 and H460 cell lines with two different YAP1 siRNAs (siYAP1 #1 and siYAP1 #2). Both YAP1 siRNAs reduced YAP1 mRNA level and protein level significantly, as shown by Q-PCR and western blot analysis (Figure 2A–2D). Knockdown of YAP1 decreased ABCG2 mRNA and protein levels. Since the two YAP1 siRNAs had similar knockdown effects, we only chose siYAP1 #2 for SP assay analysis and sphere formation analysis. SP analysis showed that knockdown of YAP1 reduced the percentage of SP cells from 1.92% to 0.735% in A549 cells and from 3.95% to 1.24% in H460 (Figure 2E to 2H). Knockdown of YAP1 also significantly reduced the number of spheres in H460 and A549 (Figure 2I and 2J).

### Overexpression of YAP1 increases ABCG2 expression and the percentage of SP cells in A549 and H460 cells

**Figure 3:**
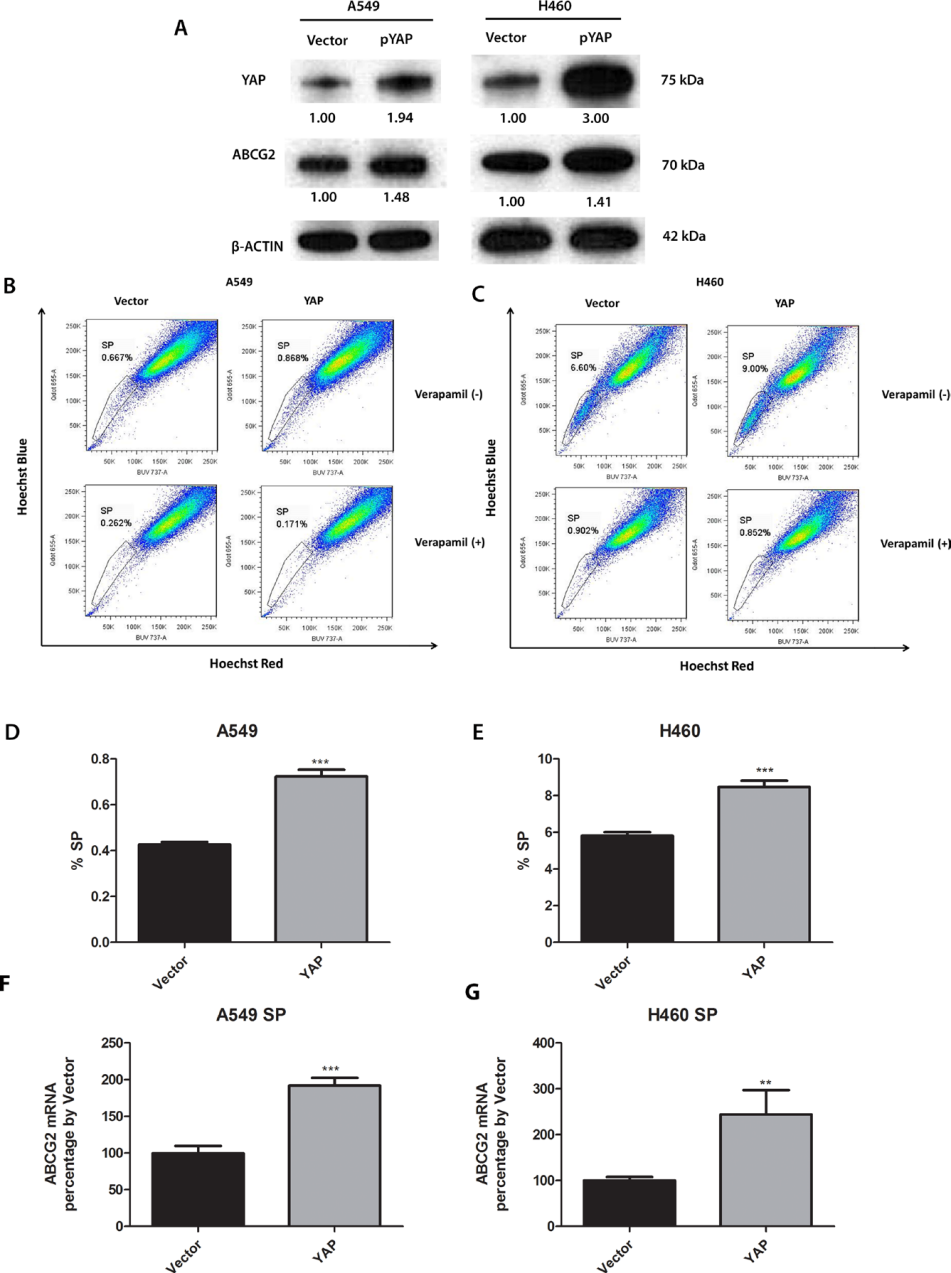
Knockdown of YAP1 decreases ABCG2 expression, the percentage of SP cells and the number of spheres formed in A549 and H460 cells (**A**) Western blot analysis of protein level of YAP1 and ABCG2 in H460 and A549 cells after YAP1 plasmid transfection. β -ACTIN was detected as a loading control. Band intensity was analyzed with ImageJ software and normalized with the intensity of β-ACTIN band. (**B–C**) Flow cytometry analysis of the percentage of SPs in A549 and H460 cells after YAP1 plasmid transfection. (**D–E**) Bar graph showing the percentage of SP cells in A549 and H460 cells after YAP1 plasmid transfection. (**F–G**) qPCR analysis of mRNA level of ABCG2 in purified SP cells of H460 and A549 after control or YAP1 plasmid transfection. Data are representative of at least three independent experiments. Error bars indicate standard deviation of triplicate SP assay data. **P < 0.005, ***P < 0.001.

To verify that ABCG2 can be regulated by YAP1 expression, we analyzed ABCG2 protein level after forced over-expression of YAP1 gene in A549 and H460 by plasmid transfection. We found that YAP1 protein level was increased after YAP1 plasmid transfection, indicating that YAP1 plasmid transfection was successful and YAP1 was overexpressed. Along with the YAP1 overexpression, ABCG2 protein level was increased (Figure 3A). The mRNA level of ABCG2 was also increased in purified SP cells after YAP1 overexpression (Figure 3F and 3G). SP assay analysis of the cells transfected with YAP1 O/E plasmid and empty vector indicated that YAP1 overexpression upregulated the SP cell portion in A549 from 0.667% to 0.868% and upregulated the SP cell portion in H460 from 6.60% to 9.00% (Figure 3B to 3E).

### Overexpression of YAP1 does not increase ABCG2 expression and the percentage of SP cells in purified A549 and H460 non-SP cells

**Figure 4:**
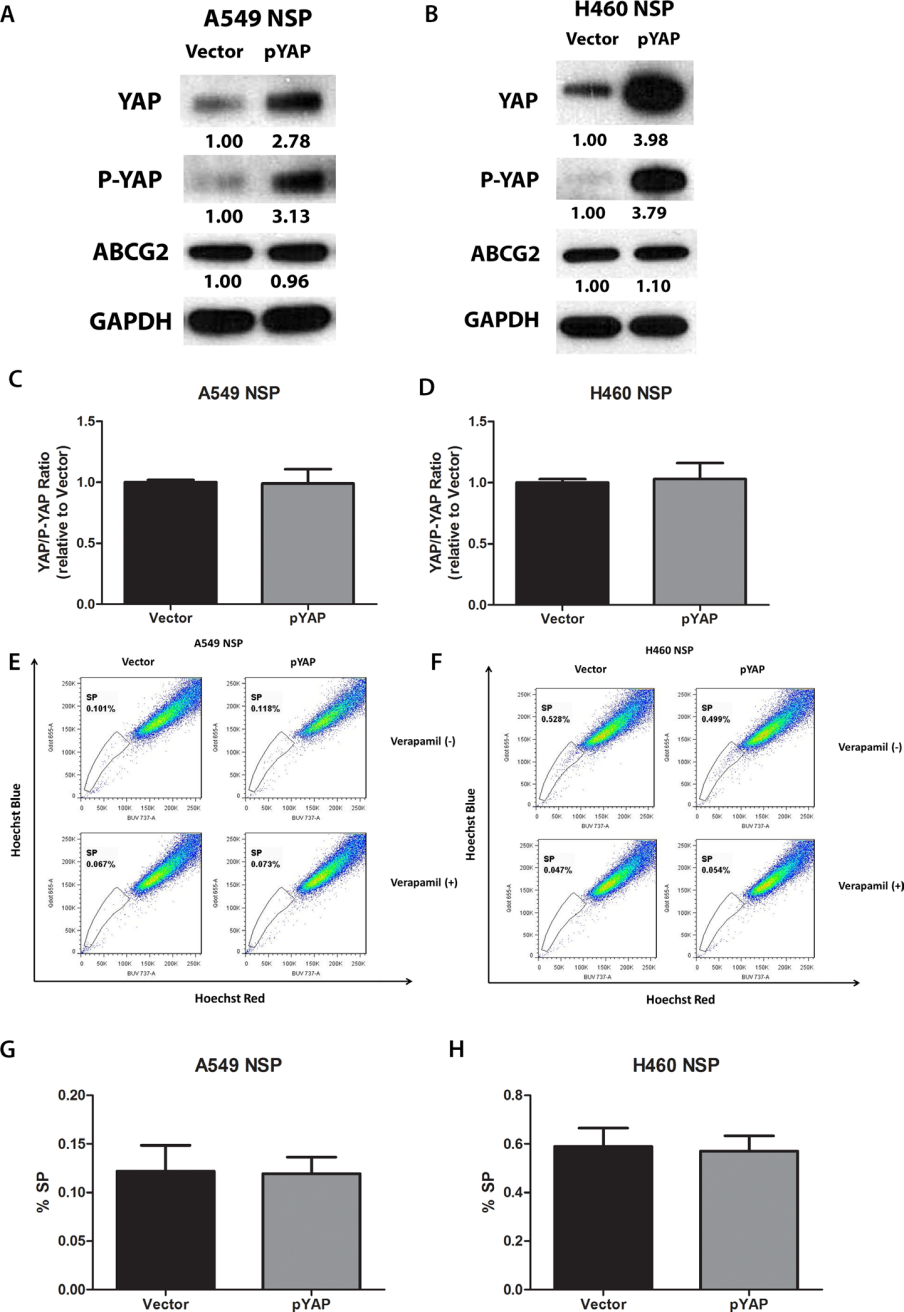
Overexpression of YAP1 does not increase ABCG2 expression and the percentage of SP cells in purified A549 and H460 non-SP cells (**A–B**) Western blot analysis of protein levels of YAP1, P-YAP1 (Ser127) and ABCG2 in A549 and H460 cells after YAP1 plasmid transfection. GAPDH was detected as a loading control. Band intensity was analyzed with ImageJ software and normalized with the intensity of GAPDH band. (**C–D**) Bar graph showing YAP/P-YAP ratio in purified non-SP (NSP) of A549 and H460 after YAP1 plasmid transfection. (**E–F**) Flow cytometry analysis of the percentage of SPs in A549 and H460 cells after YAP1 plasmid transfection. (**G–H**) Bar graph showing the percentage of SP cells in A549 and H460 cells after YAP1 plasmid transfection. Data are representative of at least three independent experiments.

To examine whether YAP1 can actively turn non-SP cells into SP cells, we purified non-SP cells from A549 and H460, over-expressed YAP1 through YAP1 plasmid transfection, and measured the change in the percentage of SP cells. YAP1 protein level was increased nearly 3-fold after transfection, which indicated YAP1 was successfully overexpressed (Figure 4A and 4B). However, ABCG2 protein level and the percentage of SP cells did not increase after overexpression of YAP1 in purified non-SP cells (Figure 4E to 4F). Since YAP1 activity was lower in H460 non-SP cells due to higher level of active LATS1, we wondered if the unchanged ABCG2 level and SP percentage were due to inactivation of YAP1 by phosphorylation on Ser 127 of YAP1. We examined the phosphate-YAP1 (S127) level and found an increase of P-YAP1 together with the increase of total YAP1. Comparing the YAP1/P-YAP1 ratio, we found no difference between control and YAP1- overexpressed non-SP cells (Figure 4C and 4D). However, when we overexpressed YAP1 S127A, the YAP1 mutant that cannot be phosphorylated by LATS1/2 and is continuously active, the SP percentage of purified non-SP cells was increased (Supplementary Figure S1A–S1D). When we overexpressed YAP1 wild type in purified and cultured SP cells, the SP percentage also was increased (Supplementary Figure S2A–2D).

### YAP1 regulates ABCG2 at the transcriptional level through binding to the promoter of ABCG2

**Figure 5:**
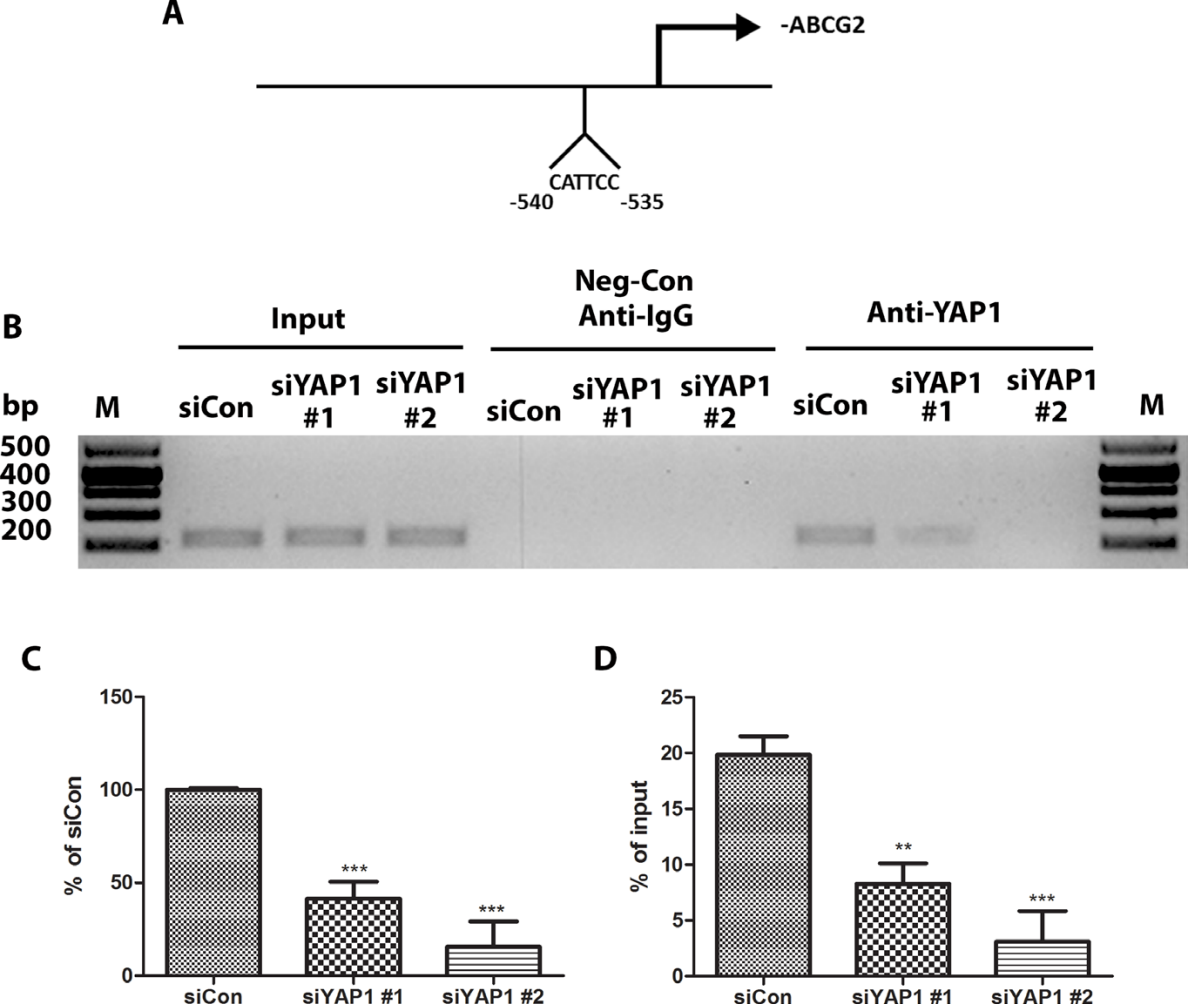
YAP1 regulates ABCG2 at the transcriptional level through binding to the promoter of ABCG2 (**A**) Schematic of the ABCG2 promoter region. Sequence analysis revealed a putative YAP1-TEAD co-binding site between −535 to −540 nucleotides upstream of the transcription start site. (**B**) ChIP assays were performed with H460 cells transfected with control siRNA (siCon) and two YAP1 siRNAs. (C) Bar graph showing band intensity of gel band of RT-PCR products. (**C–D**) qPCR analysis of ChIP assay product from H460 cells transfected with control siRNA (siCon) and two YAP1 siRNAs. Data are representative of at least three independent experiments. Error bars indicate the standard deviation of triplicate ChIP and qPCR data. **P < 0.005, ***P < 0.001.

Our earlier experiments indicated that ABCG2 expression is regulated by YAP1. YAP1 as a transcription coactivator, together with TEAD family proteins, regulates many genes, including CTGF [14]. We therefore wondered whether YAP1 regulates ABCG2 at the transcriptional level. We examined the ABCG2 promoter region (−1000 bp upstream of transcription starting site of ABCG2) and found one putative TEAD-binding site (CATTCC), which is 540 bp upstream of the ABCG2 transcription start site (Figure 5A). We used chromatin immunoprecipitations (ChIPs) to test our hypothesis. We found that in H460 cells, ChIP studies using a YAP1-specific antibody resulted in the precipitation of ABCG2 promoter region encompassing the putative TEAD binding site (Figure 5B). In the control ChIP assay using Rabbit IgG or without any antibody, we did not detect ABCG2 promoter-region binding. These findings confirmed the direct occupation of YAP1 to the promoter region of ABCG2. Performing ChIP on YAP1 siRNA transfected samples, we detected less ABCG2 promoter region precipitation in H460 cells (Figure 5B and 5C). The results of our Q-RT-PCR experiments also confirmed that YAP1 siRNA decreased YAP1 transcriptional regulation activity on ABCG2 (Figure 4D). We also found that TEAD1 bound to the same promoter region of ABCG2 as YAP1 (Supplementary Figure S3).

### The YAP1-TEAD complex inhibitor verteporfin reduces ABCG2 expression, SP cell percentage and sphere formation in A549 and H460 cells

**Figure 6:**
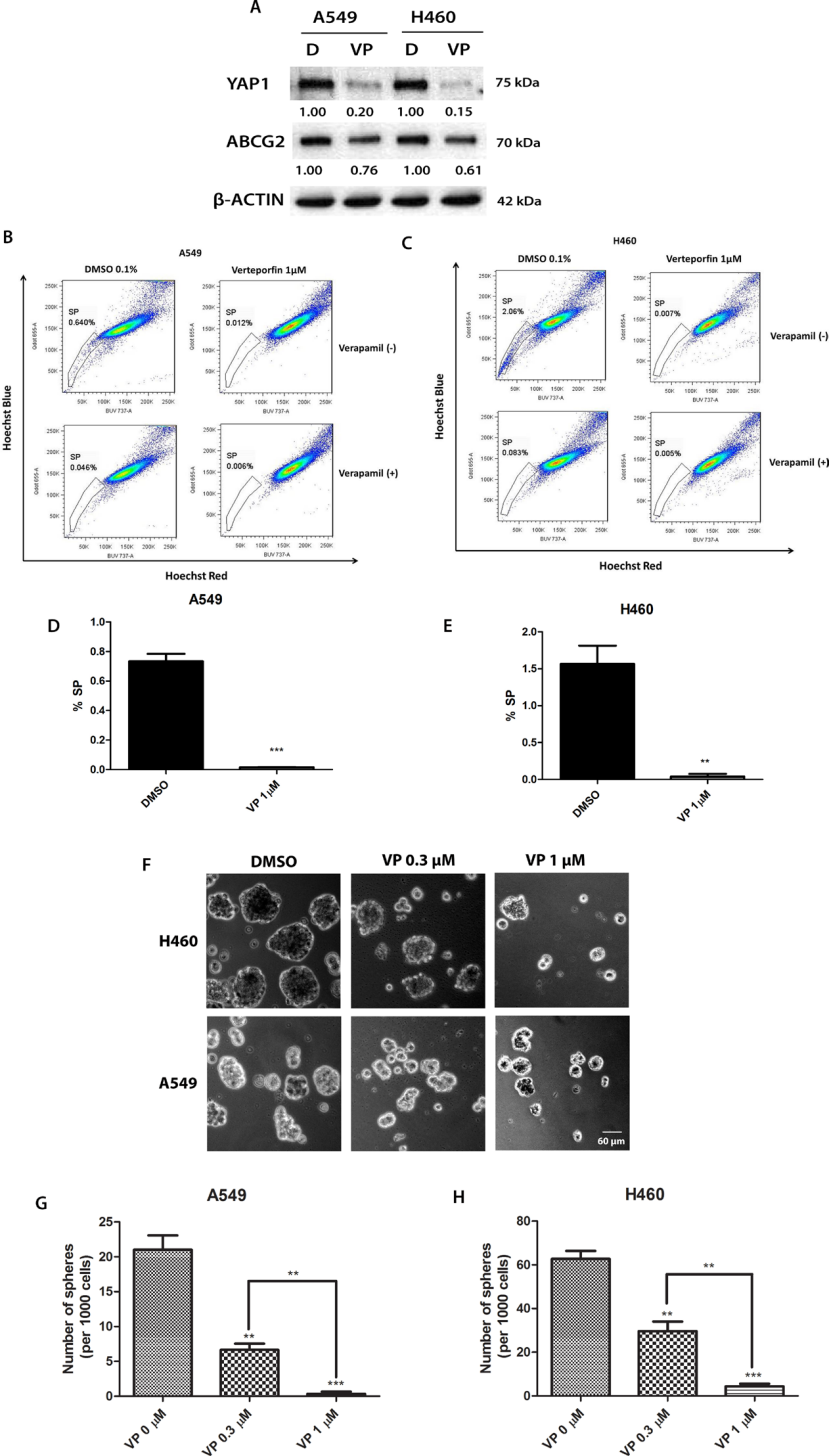
The YAP1-TEAD complex inhibitor verteporfin (VP) reduces ABCG2 expression, the percentage of SP cells and sphere formation in A549 and H460 cell lines and potentiates the cytotoxicity of doxorubicin (DOX) (**A**) Western blot analysis of protein level of YAP1 and ABCG2 in H460 and A549 cells after 1 μM verteporfin treatment. β-ACTIN was detected as a loading control. Band intensity was analyzed with ImageJ software and normalized with the intensity of β-ACTIN band. (**B–C**) Flow cytometry analysis of SP cell portion in A549 and H460 cells after 1 μM verteporfin. (**D–E**) Bar graph showing SP cell portion in A549 and H460 cells after 1 μM verteporfin. (**F**) Sphere formation analysis of H460 and A549 cells after 0.1% DMSO, 0.3 μM verteporfin or 1 μM verteporfin. (**G–H**) Bar graph showing the number of spheres formed in A549 and H460 cells after 0.1% DMSO, 0.3 μM verteporfin or 1 μM verteporfin. Data are representative of at least three independent experiments.

Verteporfin is a YAP1-TEAD complex inhibitor that can inhibit the transcriptional activity of the YAP1-TEAD complex by preventing YAP1 and TEAD interaction [23–25]. To investigate whether inhibiting YAP1-TEAD activity affects ABCG2 in A549 and H460 cell lines, we treated the cells with serial concentration dilutions of verteporfin in 96 wells to measure the IC_50_ (the drug concentration that kills 50% of cells) for A549 and H460 cells and determined the concentration for the SP assay. The IC_50_ of vertepofin was 5.59 ± 1.50 μM for A549 and 5.01 ± 1.24 μM for H460. The lowest concentration of verteporfin tested whereby cells started to show a response was 1 μM. After cells were treated with 1 μM verteporfin, cell viability was 97% for A549 cells and 93% for H460 cells. The cell viabilities for the same cell lines treated with vehicle DMSO were both 100%. Therefore, we chose 1 μM as the verteporfin concentration for western blot analysis and the SP assay. The results of the western blot analysis showed that verteporfin decreased the YAP1 protein level (Figure 6A). The ABCG2 protein level also decreased when YAP1 was downregulated after verteporfin treatment (Figure 6A). We used β–actin as the protein gel loading control in the western blot analysis. SP assay analysis of the cells treated with verteporfin or vehicle DMSO indicated that verteporfin reduced the percentage of SP cells in A549 from 0.640% to 0.012% and reduced the percentage of SP cells in H460 from 2.06% to 0.007% (Figure 6B to 6E). Verteporfin also significantly reduced the sphere formation of H460 and A549 (Figure 6F to 6H).

### Verteporfin potentiates the cytotoxicity of doxorubicin

**Figure 7:**
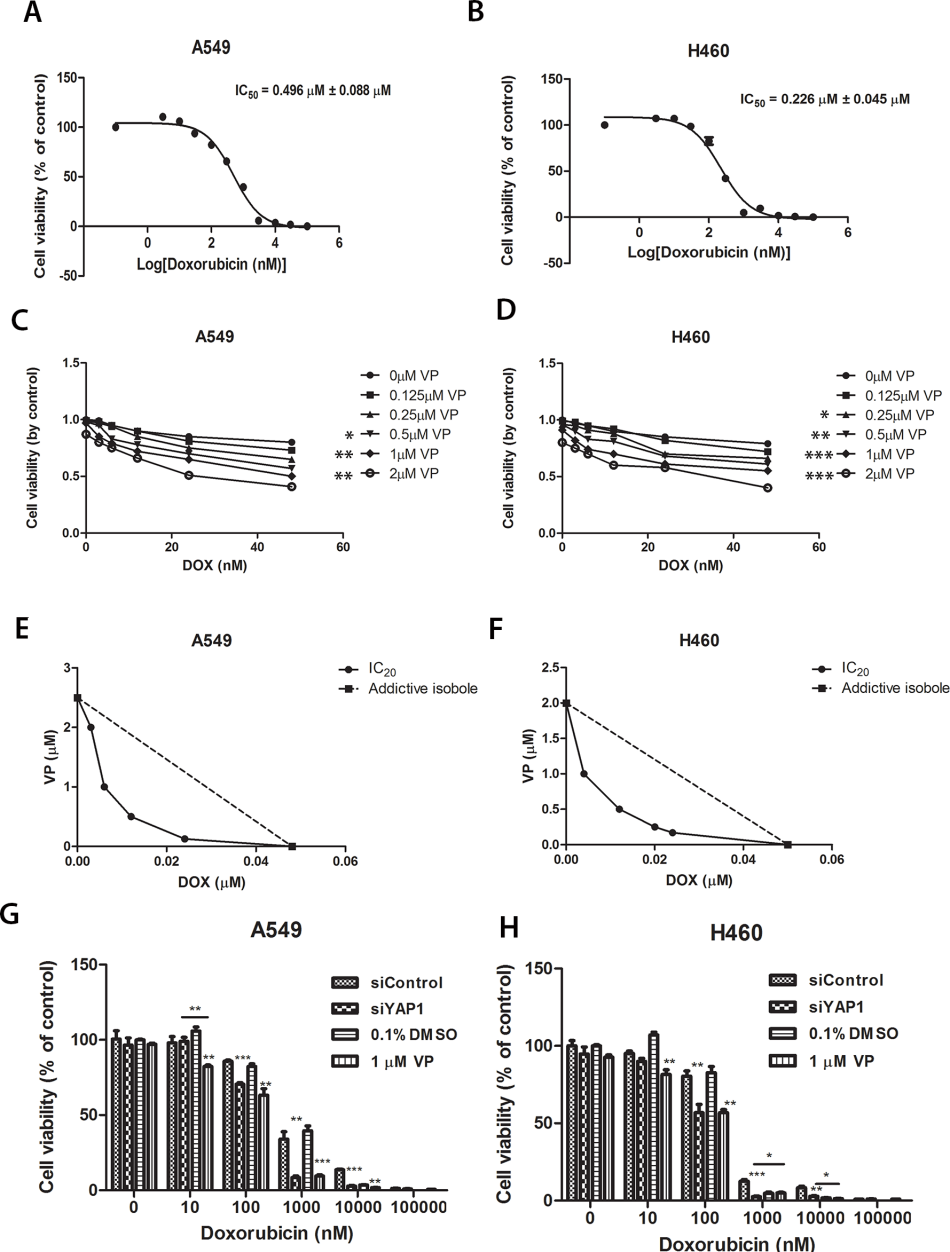
Cell viability analysis of A549 and H460 cells treated with increasing concentrations of doxorubicin combined with different concentrations of verteporfin or with siYAP1 treatment (**A–B**) Cell viability analysis of A549 and H460 cells treated with increasing concentrations of doxorubicin. Dose-dependent curve and IC_50_ were analyzed with Prism software. IC_50_s were displayed on the figures. (**C–D**) A549 and H460 cells were treated with various combinations of verteporfin and doxorubicin for 72 hours. Cell viability was determined by Titer-Glo assay. Two way ANOVA analysis was performed between the VP combination treatment group and the control group without VP addition. (**E–F**) Synergism of proliferation inhibition of A59 and H460 cells was determined by isobologram analysis. (**G–H**) Cell viability of A549 and H460 with increasing concentrations of doxorubicin combined with siYAP1 or 1 μM verteporfin. Data are representative of at least three independent experiments. *P < 0.05, **P < 0.005, ***P < 0.001.

ABCG2 is involved in multidrug resistance including to doxorubicin [26]. Since verteporfin can inhibit ABCG2 transcription, we asked whether verteporfin could help doxorubicin inhibit tumor cell growth. We measured doxorubicin IC_50_ in A549 and H460 cells (Figure 7A and 7B). The IC_50_ of doxorubicin was 0.496 ± 0.088 μM for A549 cells and 0.226 ± 0.045 μM for H460 cells. When the cells were treated with doxorubicin alone or together with of various concentrations of verteporfin for 72 hours, we found that the combination of doxorubicin and verteporfin yielded stronger cytotoxic effects than either drug alone (Figure 7C and 7D). The isobolograms of IC_50_ were used to measure the combination index (CI) of two drugs [27] (Figure 7E and 7F). The IC_50_ isoboles lay to the left of the additive isoboles, which indicated synergistic action (CI < 1), which in turn indicated that verteporfin significantly potentiated the cytotoxicity of doxorubicin in A549 and H460 cells. We also examined the effect of siYAP1 on the cytotoxicity of doxorubicin in A549 and H460 cells. Transfection of siYAP1 alone did not significantly reduce cell viability (Figure 7G and 7H) in either cell line. However, siYAP1-transfected cells were more sensitive to doxorubicin at different concentrations. The enhancement of doxorubicin cytotoxicity by siYAP1 and 1 μM verteporfin was similar (Figure 7G and 7H). The IC_50_ of doxorubicin with 1 μM of vertoporfin was slightly higher than that of doxorubicin with siYAP1 (Supplementary Table S1A). The IC_50_ of doxorubicin was lower with 2 μM of verteporfin than with siYAP1 (Supplementary Table S1A). We also compared the sensitivity of SP and non-SP cells to doxorubicin with and without 1 μM verteporfin (Supplementary Table S1B). The higher IC_50_ of doxorubicin in SP cells than in non-SP cells both in A549 and H460 cell lines indicated SP cells were more resistant to doxorubicin toxicity. Addition of 1 μM verteporfin with doxorubicin reduced the IC_50_ of doxorubicin in SP cells and in non-SP cells.

## DISCUSSION

The Hippo pathway consisting of the tumor-suppressing core-kinase cascade (MST and LATS) and oncogenic downstream effector YAP1/TAZ participates in tissue homeostasis, organ size control, and tissue repair and regeneration by regulating tissue-specific stem cells [18]. Dysregulation of the Hippo pathway leading to abnormal activity of YAP1/TAZ (e.g., a high protein level of YAP1/TAZ and/or low phosphorylation level by LATS) has been associated with cancer development. Moreover, compelling evidence supports a role of YAP1/TAZ in cancer stem cells. Our study provides several lines of evidence for an expanded role for YAP1 in lung SP cells or cancer stem-like cells. First, YAP1 is overexpressed in purified H460 SP cells compared to non-SP cells. Further contributing to YAP1 activation, phosphorylation of S127 in YAP1 is also decreased in SP cells, indicating a hyper-activation status of YAP in SP cells. Second, ectopic expression of YAP1 increased the percentage of SP cells in both A549 and H460. Conversely, knockdown of YAP1 by siRNA or reducing YAP1/TEAD transcriptional activity by the YAP1 inhibitor verteporfin decreased the percentage of SP cells and cell self-renewal activity to form spheres in A549 and H460 cells. Third, overexpression of YAP1 increased the percentage of SP cells in purified H460 SP cells. However, WT YAP1 overexpression cannot convert purified non-SP cells into SP cells because the percentage of SP cells was similar for control and YAP1 O/E cells. In contrast, overexpression of YAP-S127A, which lacks the phosphorylation site required for inactivation by the Hippo pathway, can convert NSP cells to SP cells and increase the percentage of SP cells in purified H460 non-SP cells. This is consistent with our finding that protein and phosphorylation levels of LATS1 were lower in non-SP cells than in SP cells. The upper signal contributing to this difference is unknown. It may be regulated by a canonical Hippo pathway like MST1/2 or NF2 [28], or by Rho-GTPase independent of the Hippo pathway [29]. Interestingly, TAZ expression did not differ between H460 SP and non-SP cells. This is different from the breast cancer scenario. In breast cancer stem cells, TAZ, not YAP1, has been shown to be a key regulator of breast cancer stem cells. Collectively, our findings suggest an important role of the Hippo pathway and YAP1 in lung cancer stem-like cells.

We observed spontaneous conversion of non-SP-to-SP and SP-to-non-SP (Supplementary Figure S4). The conversion of non-SP-to-SP under our normal lung cancer cell culture conditions is slower than the conversion of SP-to-non-SP. But both the purified SP and non-SP cells reached a steady level resembling that of unsorted cultures after limited culture days and limited passages. Although the 4-way purity setting we used in a FACS-ARIA II cytometer is supposed to only sort drops free of contaminating particles, we cannot rule out possible cross-contamination between SP and non-SP fractions during cell purification. The reversibility of SP and non-SP was also found in embryonic stem cell cultures [30]. Despite use of a single cell purification method, there was still a conversion between these two fractions. Studies using other stem cell markers to purify stem cells also found that normal and neoplastic non-stem cells can spontaneously convert to a stem-like state [31]. The mechanism underlying the reversibility of SP and non-SP is currently unknown.

SP cells in human cancer cells show stem-like properties, including high tumorigenic activity and chemo-resistance [8, 9]. ABCG2 is the major contributor of the SP cell properties of lung cancer cells [5, 6, 8]. It is partially responsible for multi-drug resistance to chemotherapeutic treatment and its overexpression is linked to adverse prognosis in common forms of cancer [3, 4, 11, 26, 32–36]. We found co-localization of ABCG2 and the Hippo pathway effector YAP1 in lung carcinoma SP cells. Our ChIP data indicated that YAP1 directly regulated ABCG2 expression at the transcriptional level in H460 lung cancer cells. The change in ABCG2 protein level was concurrent with YAP knockdown or overexpression, which also suggested a positive regulation of ABCG2 by YAP1. Hence addition of a YAP inhibitor to an ABCG2 substrate like doxorubicin [37] is highly likely to cause a synergetic rather than an additive effect on cancer cell viability. Consistently, we found that co-treatment with the chemotherapeutic agent doxorubicin and the YAP1-TEAD inhibitor verteporfin, or knockdown of YAP expression with siYAP1, improved the efficacy of doxorubicin in lung carcinoma cells in a synergistic manner. This synergistic effect was also present in purified SP and non-SP lung cancer cells (Supplementary Table S1B).

Accumulating evidence suggests that YAP1 plays a role in promoting drug resistance, including chemotherapy and target therapy [19–22, 38, 39]. The mechanism of YAP1-promoted resistance includes activation of the receptor tyrosine kinase *AXL*, the apoptosis inhibitor *Survivin* (*BIRC5*) genes, and autophagy depending on different drugs in different cancers. Our finding expands what is known about YAP1-promoted drug resistance through regulating a well-known ABC-transporter, ABCG2, in lung cancer. To the best of our knowledge, ours is the first study to report that YAP1 directly transcriptionally regulates ABCG2, a major multidrug transporter implicated in lung cancer, and that deactivation of YAP1 efficiently eliminates SP cells in lung cancer cells. We cannot rule out that crosstalk or a combination of different mechanisms underlies the synergistic effect of verteporfin or siYAP with doxorobucin treatment in lung cancer cells. A recent study also linked YAP1 overexpression with other ABC-transporters, including ABCC1 and ABCB1, in ovarian initiated cells [40]. However, ABCG2 was not reported as a downstream effector of YAP1 in that study.

Because YAP1 holds potential for cancer therapy, the mechanism by which the YAP1 inhibitor verteporfin exerts its action has also been studied. Verteporfin initially was proposed as a YAP1-TEAD complex inhibitor that prevented YAP1-TEAD interaction [24]. Subsequently, more evidence, including our current study, indicated verteporfin can also decrease YAP1 protein level [39]. The mechanism for YAP reduction by verteporfin is through upregulation of 14-3-3σ, which sequesters YAP1 in the cytoplasm and leads to YAP1 degradation [41]. However, verteporfin can act as an autophagosome inhibitor by promoting oligomerization of p62 and inhibit colon cancer progression independently of YAP1 [25, 42]. Hence, more specific inhibitors for YAP1/TEAD are needed for future study and clinical application.

ABCG2 has been shown to be transcriptionally regulated by Gli1, E2F1 and Nrf2 under different conditions in different cell types [43–45]. Further study is needed to determine whether the regulation of ABCG2 by these proteins and YAP1 involves cross-talk or is independent, and which protein is the major regulator under different scenarios. YAP1 is known to play important roles in organ size control and tumorigenesis [12–14]. Our study adds an important new function for YAP1 that may be relevant to cancer stem cells, drug resistance and cancer therapy. Future studies should focus on whether YAP1-ABCG2 regulation is active in a variety of tumor types and whether this affects the outcomes of clinical drugs for each disease.

## MATERIALS AND METHODS

### Cell culture

A549 and H460 established human lung cancer cell lines (originally purchased from The American Type Culture Collection, Manassas, VA) were cultivated in RPMI-1640 medium supplemented with 10% fetal bovine serum, 100 units/ml penicillin and 100 μg/ml streptomycin. Culture flasks were kept at 37°C and 5% CO_2_ in a humidified atmosphere.

### Reagents

Verteporfin and (±)-verapamil hydrochloride were purchased from Sigma (St. Louis, MO, USA). Hoechst 33342 and propidium iodide were purchased from Invitrogen Corporation (Waltham, MA, USA). Doxorubicin hydrochloride was purchased from Fisher Scientific (Pittsburgh, KS, USA). The SMARTPool siRNAs targeting YAP1 and control siRNA were purchased from Thermo Scientific Dharmacon (Pittsburgh, PA, USA). The YAP and YAP S127A plasmid DNA were purchased from Addgene (Cambridge, MA, USA).

### Cell viability assay

The cytotoxicity of verteporfin was evaluated with the CellTiter-Glo luminescent cell viability assay (Promega, Madison, WI). Cells were seeded in 96-well plates at 5000 cells/well density, incubated for 24 hours for attachment and treated with different concentrations of verteporfin, or with doxorubicin with or without verteporfin for another 72 hours. Then 100 μl of the CellTiter-Glo reagent was added into each well for a 10-minute incubation. The plate was read by a GloMax 96 microplate luminometer (Promega, Madison, WI) to monitor the luminescence signal generated by the luciferase-catalyzed reaction of luciferin and ATP.

### Side population assay and sorting

Cells were treated with 0.1% DMSO (control) or 1 μM verteporfin for 72 hours, trypsinized and resuspended in DMEM with 2% (v/v) FBS medium at 1 × 10^6^ cells/ml concentration, and incubated with 5 μg/ml Hoechst 33343 dye in the presence or absence of 100 μM verapamil at 37°C for 60 min. Tubes were gently inverted every 20 min and then centrifuged at 400 × g for 5 min at 4°C. The pellets were resuspended in cold PBS containing 2 μg/ml propidium iodide and analyzed on a BD FACS Aria II cell sorter. Emission was collected through a 610-nm long pass dichronic mirror to a 620-nm long pass filter for the Hoechst red (x-axis) collection and a 424/44-nm band pass filter for the Hoechst blue (y-axis) collection. The side population was identified as a group of cells able to exclude the Hoechst dyes, a characteristic inhibited with verapamil. In each experiment, the SP gate was set on the basis of the 100 μM verapamil control sample. The detailed step-by-step gating strategy to exclude debris and dead cells was followed [46]. Side population cells and non-SP cells were sorted into 5 ml tubes. The sorting mode was set as Device: 2 tubes; Precision: 4-Way Purity; Target Events: Continuous.

### Sphere formation assay

Cells were treated with 0.1% DMSO (control) or with 0.3 or 1 μM verteporfin for 72 hours. Cells were then trypsinized and resuspended in serum-free α-MEM supplemented with 20 ng/ml EGF, 10 ng/ml bEGF, and B27 (BD Biosciences, San Jose, CA) and 1 × 10^3^ cells /well were seeded into 24-well ultra-low adhesion plates (Corning, Corning, New York). The cells were cultured for 5 days, and then spheres with diameter larger than 50 μm in 9 fields were counted.

### Western blot analysis

Side population and non-SP H460 Cells were sorted and collected from the cell sorter, pelleted down, washed with PBS and lysed with M-PER Mammalian Protein Extraction Reagent (Thermo Scientific) supplied with Complete Protease Inhibitor Cocktails (Roche). Protein concentration was measured with a colorimetric BCA Protein Assay Kit (Pierce). 10 μg protein were separated on 4–20% precast polyacrylamide gels (BioRad) and transferred onto PVDF membranes. Membranes were blocked with 5% non-fat milk in Tris Buffered Saline-Tween (TBS-T) at room temperature for 1 hr and incubated with ABCG2 antibody (Santa Cruz Biotech, Santa Cruz, CA, USA) at 1:1000 dilution or with β-actin at 1:40000 dilution overnight followed by HRP-conjugated secondary antibodies. Immunoreactive proteins were visualized using SuperSignal West Femto Chemiluminescent Substrate (Thermo Scientific).

### Quantitative real-time-PCR

Total RNA was extracted from SP and non-SP cells using the RNeasy Mini Kit (Qiagen, Valencia, CA). The cDNA was transcribed using the iScript cDNA Synthesis Kit (Bio-Rad, Hercules, CA). The cDNA was used as a template for real-time PCR using the Applied Biosystems 7000 sequence detection system (Applied Biosystems, Foster City, CA). Expression of ABCG2 and endogenous control gene β-glucuronidase (GUSB) were detected using the commercially available primer and probe (Applied Biosystems) and analyzed using Relative Quantification Software (Applied Biosystems).

### ChIP assay

The ChIP assay was conducted using the Chromatin Immunoprecipitation (ChIP) Assay Kit (Millipore Corporation). Polyclonal antibodies for YAP (Cell Signaling Technology) and control rabbit antibody for IgG (Cell Signaling Technology) were used for ChIP. Primers used for RT-PCR to amplify the ABCG2 gene were 5′-GGTACTGATCAGCCCAATGA-3′ and 5′- TGCGACCCGGCTGAAAGCGC-3′, resulting in a product size of 202 bp. Primers used for quantitative PCR to detect ABCG2 were 5′-GGTACTGATCAGCCCAATG A-3′ and 5′-CAGGGACAAGCCAAACACT-3′. This Q-PCR analysis was performed using Qiagen SYBR Green/Rox qPCR Master Mix (Qiagen) and Applied Biosystems 7000 sequence detection system (Applied Biosystems, Foster City, CA).

### Statistical analysis

Data are presented as mean ± standard deviation (SD). Statistical significance of differences between different groups was determined by Student's *t*-test. The level of statistical significance was set at ≤ 0.05. The combination index (CI) was calculated by CompuSyn software using the Chou and Talalay method [47]. CI < 1, = 1 and > 1 represent synergy, additivity, and antagonism, respectively.

## SUPPLEMENTARY MATERIALS FIGURES AND TABLES


