# Supplementary material for: YAP1 regulates ABCG2 and cancer cell side population in human lung cancer cells
Source: Oncotarget. 2016 Nov 29;8(3):4096–109. doi: 10.18632/oncotarget.13686 (PMC5354815; doi:10.18632/oncotarget.13686)
Supplement: Supplementary file 1 [file oncotarget-08-4096-s001.pdf]

YAP1 regulates ABCG2 and cancer cell side population in human lung cancer cells

Supplementary Materials

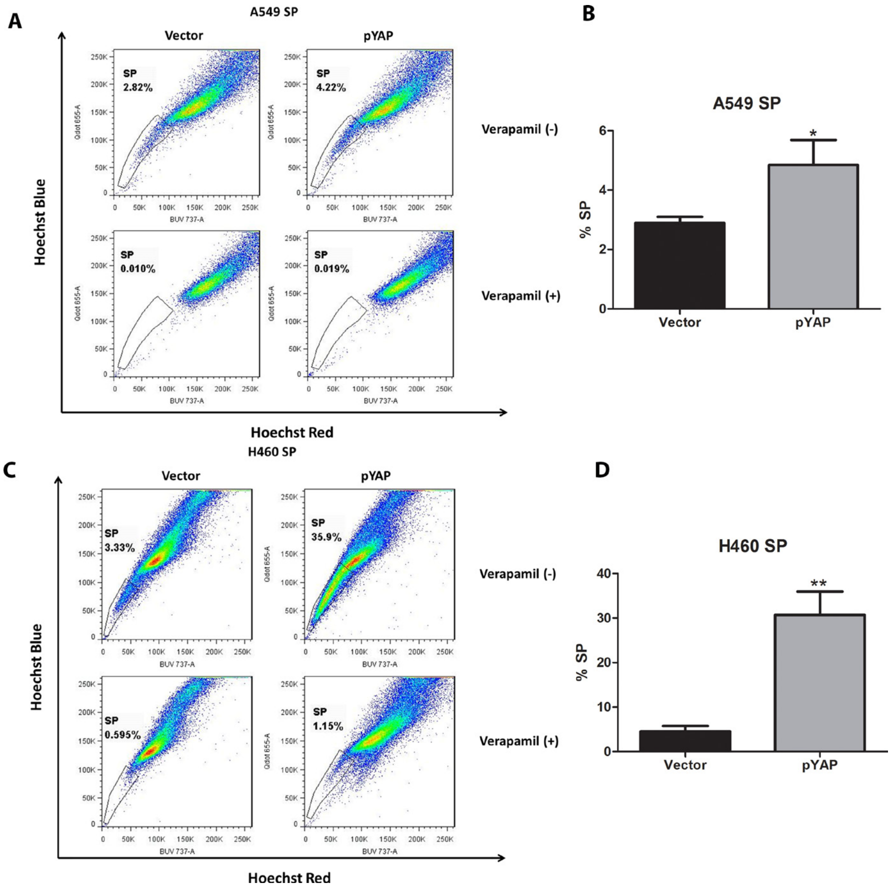

Supplementary Figure S1: (**A** and **C**) **Flow cytometry analysis of the percentage of SPs in purified and cultured SP cells of A549 and H460 cells after YAP1 plasmid transfection.** (**B** and **D**) Bar graph showing the percentage of SP cells. Data are representative of at least three independent experiments. Error bars indicate the standard deviation of triplicate SP assay data. *$P < 0.05$, **$P < 0.005$.

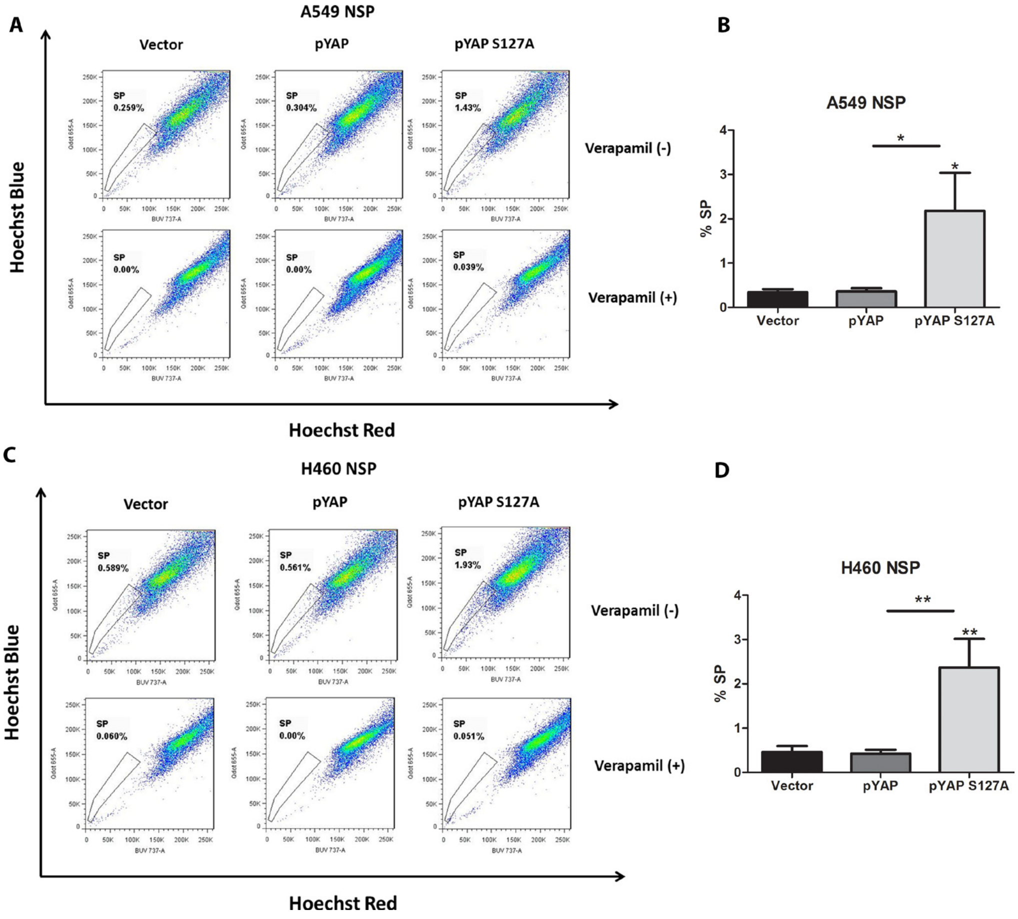

Supplementary Figure S2: (**A** and **C**) **Flow cytometry analysis of the percentage of SPs in purified and cultured non-SP cells of A549 and H460 cells after YAP1 or YAP1 S127A mutant plasmid transfection.** (**B** and **D**) Bar graph showing the percentage of SP cells. Data are representative of at least three independent experiments. Error bars indicate the standard deviation of triplicate SP assay data. *$P < 0.05$, **$P < 0.005$.

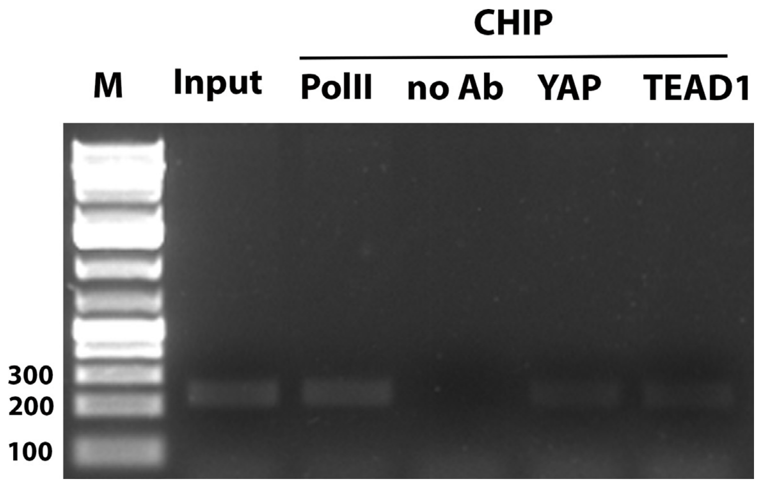

Supplementary Figure S3: ChIP assays were performed with H460 cells using YAP1 antibody and TEAD1 antibody. The sample using anti-RNA Polymerase II (Pol II) to pull down ABCG2 promoter was used as a positive control.

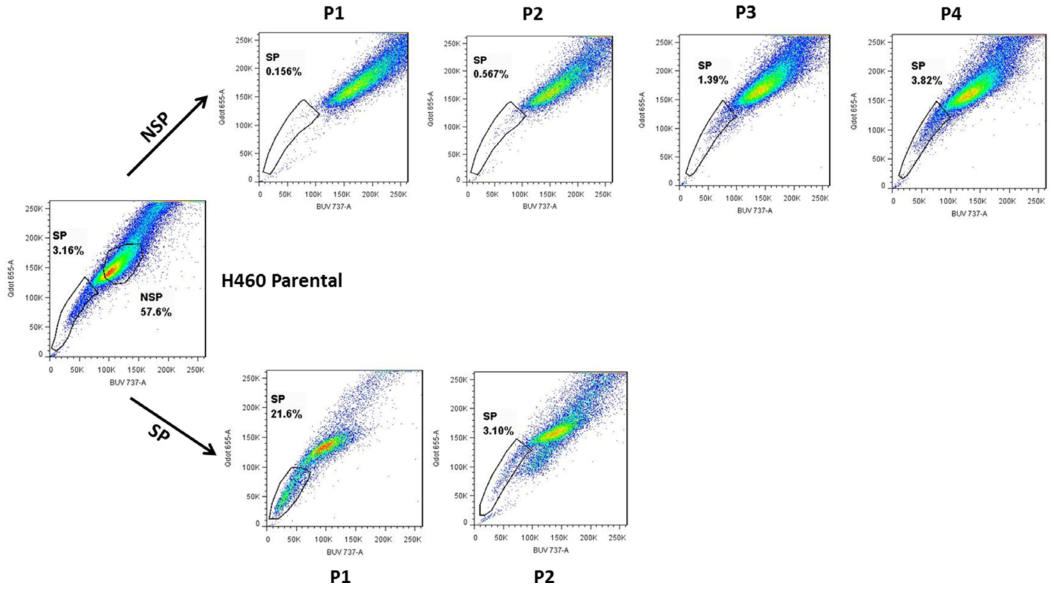

Supplementary Figure S4: Flow cytometry analysis of the percentage of SPs in purified and cultured non-SP (NSP) and SP cells of A549 and H460 cells after several passages.

Supplementary Table S1A: Sensitivity (IC_{50} µM) of A549 and H460 cells to doxorubicin with verteporfin and siYAP1

	siYAP1	doxorubicin + 1 µM verteporfin	doxorubicin + 2 µM verteporfin
**A549**	0.173 ± 0.020	0.217 ± 0.013	0.063 ± 0.010
		$P = 0.0335^*$	$P = 0.0010^*$
**H460**	0.124 ± 0.011	0.161 ± 0.014	0.095 ± 0.011
		$P = 0.0228^*$	$P = 0.0315^*$

Supplementary Table S1B: Sensitivity (IC_{50} µM) of SP and NSP cells to doxorubicin with verteporfin

	doxorubicin	doxorubicin+ 1 µM verteporfin	
**A549 SP**	1.27 ± 0.31	0.41 ± 0.15	$P = 0.012^*$
**A549 NSP**	0.37 ± 0.12	0.14 ± 0.07	$P = 0.041^*$
	$P = 0.009^*$	$P = 0.008^*$	
**H460 SP**	0.67 ± 0.08	0.23 ± 0.13	$P = 0.008^*$
**H460 NSP**	0.20 ± 0.05	0.13 ± 0.07	$P = 0.232$
	$P = 0.001^*$	$P = 0.306$	

Supplementary Table S1C: Sensitivity (IC_{50} µM) of SP and NSP cells to doxorubicin, cisplatin, and temozolomide

	doxorubicin	cisplatin	temozolomide
**A549 SP**	1.27 ± 0.31	16.66 ± 1.91	274.93 ± 16.88
**A549 NSP**	0.37 ± 0.12	13.92 ± 1.44	298.94 ± 23.65
	$P = 0.009^*$	$P = 0.11830$	$P = 0.226$
**H460 SP**	0.67 ± 0.08	5.51 ± 0.47	834.61 ± 50.28
**H460 NSP**	0.20 ± 0.05	4.08 ± 0.15	740.26 ± 30.89
	$P = 0.0010^*$	$P = 0.00730^*$	$P = 0.050^*$

Values were expressed as mean ± SD, done in triplicate in three independent experiments. IC_{50}s were measured after drug treatment for 72 hours. Statistical significance ($P < 0.05$) indicated by asterisks (*).
